# Construction of Alizarin Conjugated Graphene Oxide Composites for Inhibition of *Candida albicans* Biofilms

**DOI:** 10.3390/biom10040565

**Published:** 2020-04-07

**Authors:** Mohankandhasamy Ramasamy, Sitansu Sekhar Nanda, Jin-Hyung Lee, Jintae Lee

**Affiliations:** 1 School of Chemical Engineering, Yeungnam University, Gyeongsan 38541, Korea; mohan.ramasamy@uwaterloo.ca (M.R.); jinhlee@ynu.ac.kr (J.-H.L.); 2 Department of Chemical Engineering, Waterloo Institute for Nanotechnology, University of Waterloo, 200 University Avenue West, Waterloo, ON N2L 3G1, Canada; 3 Department of Chemistry, Myongji University, Yongin 449728, Korea; nandasitansusekhar@gmail.com

**Keywords:** adsorption, graphene oxide, alizarin, antibiofilm, *C. albicans*, hyphal inhibition

## Abstract

Biofilm inhibition using nanoparticle-based drug carriers has emerged as a noninvasive strategy to eradicate microbial contaminants such as fungus *Candida albicans*. In this study, one-step adsorption strategy was utilized to conjugate alizarin (AZ) on graphene oxide (GO) and characterized by ultraviolet-visible spectroscopy (UV-Vis), attenuated total reflectance Fourier transform infrared spectroscopy (ATR-FTIR), X-ray powder diffraction (XRD), dynamic light-scattering (DLS), and transmission electron microscopy (TEM). Crystal violet assay was performed to evaluate the antibiofilm efficacy of GO-AZs against *C. albicans*. Different characterizations disclosed the loading of AZ onto GO. Interestingly, TEM images indicated the abundant loading of AZ by producing a unique inward rolling of GO-AZ sheets as compared to GO. When compared to the nontreatment, GO-AZ at 10 µg/mL significantly reduced biofilm formation to 96% almost equal to the amount of AZ (95%). It appears that the biofilm inhibition is due to the hyphal inhibition of *C. albicans*. The GO is an interesting nanocarrier for loading AZ and could be applied as a novel antibiofilm agent against various microorganisms including *C. albicans*.

## 1. Introduction

Fungal infections are considered a major source of human disease especially in immunocompromised individuals, burn victims, neonates, and patients with serious illness [1]. Amongst them, the opportunistic eukaryotic fungal pathogen *Candida albicans* causes an unrecoverable deep tissue infection, high mortality, and socioeconomical challenge [2]. *C. albicans* is the predominant pathogen isolated from various medical devices, including catheters, joint protheses, pacemakers, contact lenses, and dentures [3]. The majority of the implantable medical devices are susceptible to *Candida* spp. biofilm formation. *C. albicans* growth comprises budding yeasts, pseudohyphae, or true hyphae. The transition of yeast cells to hyphae is considered a crucial virulence factor in *Candida* infections and appears to positively control biofilm formation [4]. The hyphae intertwined with biofilm cause pathogenicity and the transitioned hyphae from yeast cells can adhere to host cells, can damage tissues, and can escape from host immune defense system [5]. Therefore, it is necessary to develop a suitable material that can inhibit hyphae formation as well as biofilm formation.

The prevalence of antibiotic resistant microorganisms poses a worldwide problem that immediately requires novel antibiotic and non-antibiotic strategies. Lacking agents bringing out the development of potent new materials that can prevent *Candida* biofilms, especially on the implantable medical devices, are becoming more important field of research among biomaterialists [6]. In general, chemical-based antimicrobials have been primarily applied to combat biofilms. However, impregnated chemicals alone cannot withstand the harsh in vivo biological conditions and they have shown limited applicability because of the increased resistance of biofilm microorganism [7]. Hence, researchers have now focused on conjugating effective chemical compounds that are not prone to antifungal resistance, with nanomaterials for pathogenic fungal antibiofilm therapy [8,9,10,11].

Antibiofilm strategies include organic nanoparticles; inorganic nanoparticles; polymeric nanoparticles; peptide-, gas-, ion-, or drug-releasing nanoparticles; and antimicrobial surface coating with materials including gold, chitosan, and graphene [8,9,12,13,14,15]. Amongst them, graphene-based coatings are best suitable for the development of antibacterial surfaces due to their various surface-active sites such as oxygen bonds and hydroxyl, carbonyl, carboxylic, and epoxide groups which offer straight forward fabrication procedure with potent molecules to produce a new biocompatible antimicrobial nanocomposite [16]. Conjugating bioactive molecules on nanomaterials becomes a comprehensive research area due to their successful applications in the market [17]. While choosing active substances, vital factors including availability, cost, eco-friendly nature, reusability, microbial resistance, and toxicity need to be considered. Although number of plant extracts including dyes and oils were exploited for antimicrobial purpose especially for antibiofilm therapy, still, their efficacy is debatable because of rapid volatility, instability, poor aqueous solubility, limited dispersibility, adverse physiological effects, and inability to release/deliver the payloads to the target sites [18,19,20]. To solve the said complications, incorporating active molecules or drugs with the nanomaterials and utilizing them as a substrate could be an alternative solution for antibiofilm applications. Recently, our group found that alizarin and other anthraquinones at 2–10 µg/mL significantly inhibited biofilm formation of drug-resistant *Staphylococcus aureus* [21] and *C. albicans* [22].

In this study, we utilize alizarin (AZ)—a naturally available synthetic dye to be conjugated with graphene oxide (GO) to make a commercially feasible antibiofilm agent. The GO-AZ composite was characterized by ultraviolet-visible spectroscopy (UV-Vis), attenuated total reflectance Fourier transform infrared spectroscopy (ATR-FTIR), X-ray powder diffraction (XRD), dynamic light-scattering (DLS), and transmission electron microscopy (TEM). The effect of GO-AZ against the *C. albicans* biofilm was evaluated through metabolic activities and morphological changes as compared to the control AZ and untreated control. In addition, the nematode *Caenorhabditis elegans* was used as an in vivo model to confirm the antibiofilm and antivirulence properties of GO-AZ against the pathogenic *C. albicans*. 

## 2. Materials and Method

### 2.1. Strains and Culture Conditions

*Candida albicans* (*C. albicans*) (DAY 185) from the Korean Culture Center of Microorganisms (http://www.kccm.or.kr/) was used in this study. The −80 °C glycerol-preserved C. albicans was streaked on potato dextrose agar (PDA) plates, and a 48-h grown single colony was inoculated into 25 mL of potato dextrose broth (PDB) and incubated overnight at 37 °C. All other chemicals were purchased from Sigma-Aldrich (St. Louis, MI, USA).

### 2.2. Synthesis of Dye Conjugated Graphene Oxide 

Initially, GO was synthesized by Hummer’s method with modifications as reported by Sitansu et al. [23]. Next, 1 mg/mL of GO was added dropwise to 10 mg/mL of AZ with continuous stirring for 3 h in an ice bath. Unbound AZ was removed by centrifugation at 10,000 rpm for 15 min in ethanol and water for 3 consecutive times. Purified GO-AZ composites were freeze-dried, stored, and utilized for further studies. 

### 2.3. Characterizations of Nanocomposites

The structural modification of GO before and after dye conjugation was examined using UV-Visible spectrophotometer (UV-1800, Shimadzu, Japan) and attenuated total reflectance Fourier transform infrared spectroscopy (ATR-FTIR). Morphological characteristics were investigated by a high-resolution transmission electron microscopy (HR-TEM, Tecnai G2 F20, FEI, USA) at an accelerating voltage of 200 kV. Particle features including size and zeta potential were analyzed using a Zetasizer Nano ZS dynamic light-scattering (DLS) analyzer (Malvern Instruments, Malvern, UK).

### 2.4. Antifungal Evaluation

The minimum inhibitory concentrations (MICs) of AZ, GO, and GO-AZ were performed by the Clinical Laboratory Standards Institute (CLSI) microdilution method using 96-well polystyrene plates (SPL Life Sciences, Korea). *C. albicans* was inoculated at a dilution of 1:100 in PDB medium with varying concentrations of samples and incubated at 37 °C for 24 h [24]. MIC was considered the lowest concentration that inhibited at least 80% of microbial growth, which was assessed using Optizen (2120UV) UV-vis spectrophotometer at 620 nm and by colony counting.

### 2.5. Antibiofilm Assay 

To evaluate the antibiofilm activity, suspensions of GO and GO-AZ were drop-casted on a 96-well flat bottom plate by slowly drying in an air oven at 50 °C [25]. The coated wells’ biofilm inhibition efficiency was analyzed by static biofilm forming assays of *C. albicans* and was performed on 96-well polystyrene plates (SPL Life Sciences, Korea) [26]. Briefly, stationary phase cells were cultured with or without samples at varying concentrations in PDB medium without shaking for 24 h at 37 °C. After incubation, the cell growth was measured at 620 nm and the supernatant was discarded and washed three times with distilled water to remove non-adherent cells. Excess water was removed, and the plates were dried before staining with 300 μL of crystal violet (0.1%, *v/v*) for 20 min at room temperature. Stained plates were washed, and the adsorbed crystal violet was dissolved after adding 95% ethanol for 15 min. The formed biofilm was measured at 570 nm using a Thermo Scientific Multiskan EX (Thermo Fisher Scientific, Vantaa, Finland). Biofilm inhibition was quantified from six replicates, and the results are presented as the averages with standard deviations. 

### 2.6. Visualization of Biofilm Inhibition 

Confocal laser scanning microscopy (CLSM) was performed on a 96-well flat bottom plate by following the protocol as mentioned previously with modifications [11]. Biofilms with or without sample treatment were stained in a dark room with carboxyfluorescein diacetate succinimidyl ester (Invitrogen, Molecular probes Inc., Eugene, OR, USA) for 1 h and visualized at a magnification of 20× using an Ar laser with 488 nm and 500–550 nm as excitation and emission wavelengths, respectively. A Nikon Eclipse Ti (Tokyo, Japan) microscope was used to observe stained biofilms, and NIS-Elements C version 3.2 (Nikon eclipse) was used to obtain color 3D confocal images. 

### 2.7. Hyphae Inhibition Assay 

To visualize the impact of GO-AZ on pathogenic hyphae growth, we performed hyphae inhibition assay as described previously [27]. Briefly, overnight-grown yeast cell suspension in RPMI 1640 (Invitrogen, USA) medium buffered with HEPES (pH 7.3) was incubated with AZ, GO, and GO-AZ at 37 °C for 24 h with agitation (200 r/min). Fungal cells were observed under iRiSTM digital cell imaging system (Logos Bio Systems, Korea).

### 2.8. Anti-Virulence Assay Using *C. elegans* as a Host

To investigate the effects of GO-AZ on the virulence of *C. albicans*, the nematode *C. elegans* (n = 20) strain N2 Bristol CF512 fer-15(b26); fem-1(hc17) were infected with lawns of *C. albicans* on PDA plates with AZ, GO, and GO-AZ [11]. Initially, the nematodes were grown on nematode growth medium (NGM) fed with *Escherichia coli* strain OP50. Then, the synchronized adult population was collected by washing with M9 buffer and was placed on *C. albicans* lawn with or without samples. Finally, the plates were incubated at 25 °C with gentle shaking, and *C. elegans* survivability was monitored for seven days. Experiments were performed in triplicates, and the results were expressed as percentage of alive or dead worms. 

### 2.9. Statistical Analysis

Three independent experiments were conducted, and data were expressed as means ± standard deviations. The significant difference between groups were further determined using student’s t-test. The probability level of *p* < 0.05 was statistically significant. 

## 3. Results and Discussion

### 3.1. Characterizations of GO-AZ Conjugate

**Figure 1:**
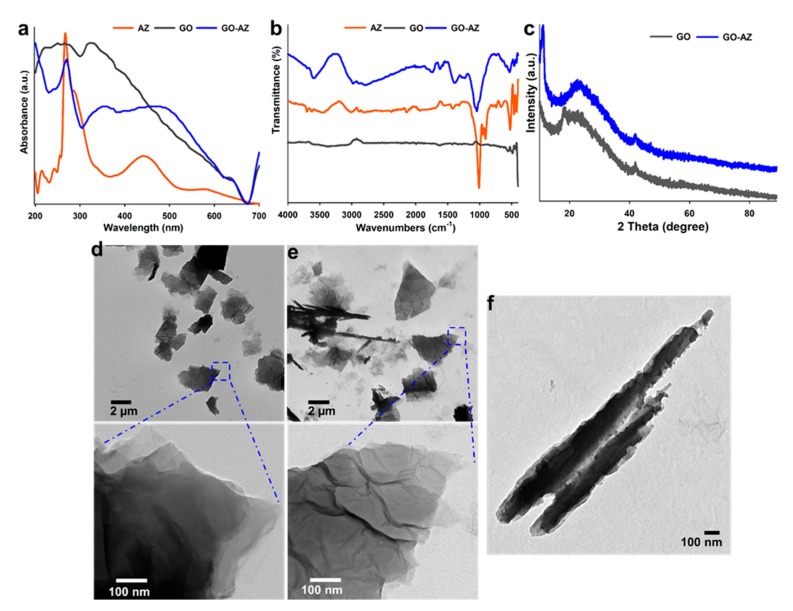
UV-Visible spectroscopy (**a**); FT-IR spectroscopy (**b**); and XRD patterns (**c**) of alizarin (AZ), graphene oxide (GO), and GO-AZ and TEM images (**d**) of GO and (**e**) GO-AZ: The lower panels show the magnified views of distinct surface changes on the GO before and after AZ conjugation (**f**) TEM image of fabricated GO-AZ nanoscroll. Obviously, visible layers of the rolls and increased contrast indicate the rolling of 2D sheets with a tightly wrapped AZ by the GO sheet.

In this study, AZ was non-covalently functionalized onto the GO by simple one-step adsorption technique [28]. UV-Visible spectroscopy was utilized to investigate the conjugation of AZ on GO because of the identification of unique absorption bands at specific wavelengths (Figure 1a). For GO, characteristic absorbance peaks were detected at ~250 for π–π* transition of C=C and ~320 nm due to n–π* transition of C=O. Pure AZ shows a band at 220 nm for π–σ* and that at 266 nm ascribed π–π* transition of the benzenoid system. Presence of shoulder peak at 280 nm and a weak signal at 330 nm assigned to quinonoid π–π* transitions. Appearance of absorption bands at longer wavelengths at 440 nm attributed to C=O of quinoid form of AZ. Further, GO-AZ showed well-resolved characteristic absorption peaks of GO and AZ in one spectrum and the signals were red shifted from their original position (GO = 320 to 350 nm and AZ = 266 to 270 and 440 to 470 nm), which confirms the successful conjugation of AZ on the surface of GO via π–π stacking interaction. 

Figure 1b showed the ATR-FTIR spectra of GO before and after surface modification with AZ. GO displayed weak IR signals around 3300 and 1630 cm^−1^, corresponding to the stretching vibrations of –OH and sp2-hybridised C=C, confirming the formation of GO sheets from substantial oxidation of graphite layers [29]. FTIR spectra of AZ and GO-AZ clearly showed a strong similarity in their unique signature bands, indicating the presence of AZ molecule on the GO (Figure 1b). The distinctive peaks at around 1050 cm^−1^ responsible for C=O stretching of the quinonyl group of AZ and the bands at 1288 cm^−1^ correspond to the aromatic C–O skeletal vibration and COH in-plane bending vibration signals at 1389 cm^−1^ [30]. The XRD patterns of GO and GO-AZ are shown in Figure 1c. Both GO and GO-AZ show broad reflection peaks, exhibiting the stacking of exfoliated graphene with poorly ordered c-axis direction. Compared with GO, the peak of GO-AZ red-shifted from 22.60° to 23.24°, which corresponding to a decrease in layer spacing because of AZ that relatively increased the peak intensity. All these results are in good agreement with the reported literature and convince of the formation of GO-AZ attempted for biomedical applications.

Morphology of the GO before and after AZ conjugation was assessed using TEM (Figure 1d,e). Well-dispersed GO sheets (Figure 1d) were formed due to the electrostatic repulsion of its abundant functional groups with the thickness about 5 nm. The conjugation process helped to attach AZ molecules through hydrophobic interaction with GO. The conjugated structure of GO-AZ (Figure 1e) provided increased π–π stacking sites to overlap at the edges of each sheets [31]. Magnified TEM results show that the formation of more wrinkles on the GO-AZ is completely different from the smooth and flat GO sheets (Figure 1d,e, lower panel), indicating a surface modification by AZ conjugation. An interesting structural phenomenon of formation of rolls was observed in the GO-AZ sheets with lengths from 2 to 5 µm with uniform diameter of 200 nm (Figure 1f). The rolls are uniquely present throughout the TEM grid, while the roll was entirely absent in GO sheets (Figure 1d,e). Elsewhere, available reports demonstrated the rolling behavior of GO sheets with the presence of inorganic nanoparticles or application of high physical forces including sonication [32]. However, this study did not use any nanoparticle or strong sonication techniques like probe sonication, indicating that the high conjugation of organic dye AZ on the active sites of GO is responsible for the formation of GO-AZ rolls. We speculate that the proper chemical reduction of graphite produced more single sheets which enhanced AZ anchoring resulting in the folding of sheets to rolls. Although there are many proposed theories available for this phenomenon, still, the exact mechanism is yet to be understood [33].

The hydrodynamic radii of GO and GO-AZ were 2300 ± 1101 to 2600 ± 1025 nm, respectively. DLS measurements of GO-AZ were significantly larger than GO, possibly due to the high incorporation of AZ on the surfaces. Likewise, surface charge of GO was −2 ± 1 mV, and after surface modification, it increased to −23 ± 6 mV for GO-AZ. The increased zeta potential confirms the AZ conjugation and gives a moderate electrostatic stability for GO-AZ in water, which is characterized by a slow sedimentation with negligible particle size change.

### 3.2. Antifungal and Antibiofilm Activities of GO-AZ Against *C. albicans*

The antifungal activity of the samples was evaluated by measuring the MIC. For AZ, GO, and GO-AZ, the MICs were 1000, >5000, and 1000 µg/mL, respectively. The MIC result of AZ matches with the previous report [22]. There was no significant difference in antifungal activity of AZ and GO-AZ.

**Figure 2:**
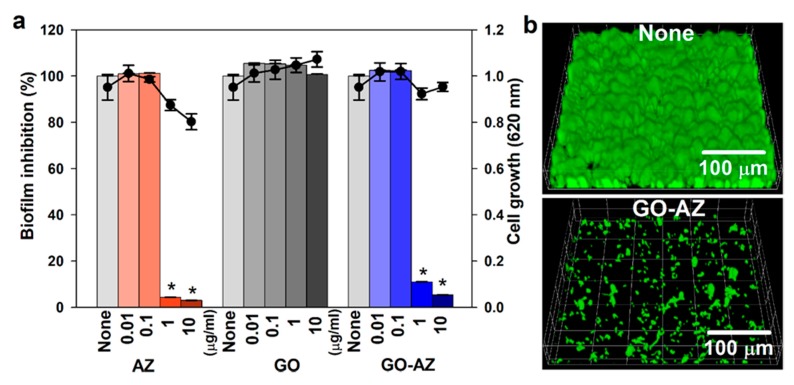
Antibiofilm activity of AZ, GO, and GO-AZ at 0.01–10 µg/mL against *C. albicans* incubated 24 h in 96-well plates without shaking (**a**): Bar data indicate biofilm formation, and lines indicate planktonic cell growth. Representative 3D confocal laser scanning microscopy observation of *C. albicans* biofilm after incubation with GO-AZ (**b**): Biofilm formation without GO-AZ was taken as none. The microscopy image shows significant biofilm inhibition in the presence of GO-AZ at 5 µg/mL compared to untreated *C. albicans*. * *p* < 0.05 vs none.

Since biofilm formation is a responsible mechanism of high resistance to antifungal drugs and grown matured biofilms can produce substantial persister cells [34], *C. albicans* can form biofilms on biomedical implants posing, a major healthcare issue associated with pathogenic fungus, and it is more difficult to eradicate than bacterial biofilm infections [35]. Because of *C. albicans* resistance to all the available antifungal drugs [36,37,38], there is an urgent need for novel strategies to alternative solutions for the prevention and treatment of *C. albicans* biofilm infection. In this work, we investigated the effects of AZ, GO, and GO-AZ on *C. albicans* biofilm formation. We evidenced the inhibition of biofilm formation (>95%) of fungal cells in the presence of increasing AZ concentration up to 10 µg/mL, (Figure 2a). Interestingly, even at higher concentration (10 µg/mL), GO maintained the biofilm growth, explaining that there was no bioactive group present. On the other hand, GO-AZ exhibited significant antibiofilm activities of 89% and 96% at 1 and 10 µg/mL like for AZ (>95%), thus confirming that AZ molecule conjugated on GO exerted the biofilm inhibition activity. There was a negligible difference in the planktonic cell growth observed between AZ and GO-AZ, claiming that AZ effectively reduced the biofilm formation of *C. albicans* without antifungal activity (Figure 2a). It is important to find an antibiofilm agent without impacting planktonic cell growth, which is less responsible for the development of drug resistance by microorganisms since biofilm formation is a mechanism of drug resistance [39]. CLSM images of untreated *C. albicans* showed a formation of compact and dense biofilms, whereas GO-AZ treatment at 5 µg/mL dramatically inhibited the biofilm thickness and densities by more than 95% than untreated control (Figure 2b). The drop-cast GO-AZ present at the bottom of the plate could be responsible for this specific antibiofilm activity due to the direct exposure of the biofilm cells to AZ, thus preventing the formation of sessile communities of *C. albicans*. Establishing a biofilm in the self-produced energy-rich extracellular polymeric matrix, signaling across the fungi cell network would promote the planktonic cells to surface-attached cells for further proliferation. In order to develop the network, a foothold by the fungi on the surface is necessary to mediate cell-to-surface and cell-to-cell interactions to develop a structural scaffold. If the surface is non-supportive or antiadhesive for the growth of biofilm, it will act as a key factor preventing undesirable fungal colonization. From our results, GO and GO-AZ surfaces differed significantly in terms of wrinkle formation and nanoscrolls in their physical front. However, AZ conjugation might not provide favorable surface properties for anchoring fungi, thereby inhibiting the cell-surface interaction and inhibiting biofilm formation.

### 3.3. Antibiofilm Mechanism of GO-AZ via Hyphae Inhibition

**Figure 3:**
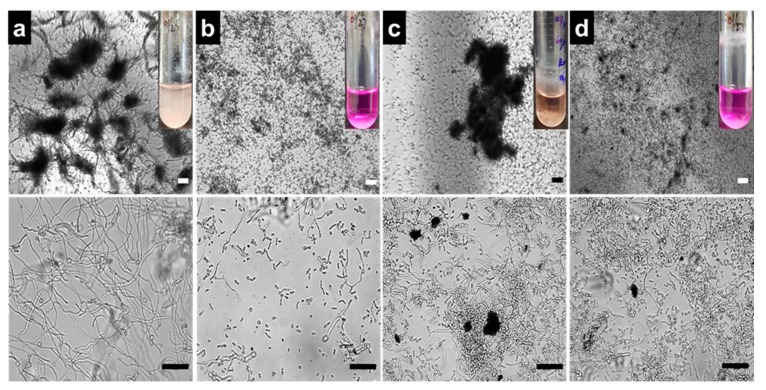
Light microscopy images of *C. albicans* hyphae formation: Micrographs showing denser filament formation in the presence of none (**a**), complete inhibition of hyphae by AZ at 2 µg/mL (**b**), slow hyphae development in the presence of 2 µg/mL GO (**c**), and hyphae inhibition in the presence of 2 µg/mL GO-AZ (**d**) with high yeast cell number. Insets show the color change of the medium from pink to colorless which exhibit the yeast-to-hyphae transition without or with samples. Lower panels showing the magnified views of the same. Scale bar = 50 µm.

The yeast-to-hyphae transition and formation of cell aggregates are prerequisites of biofilm development by *C. albicans* [40]. To examine the effects of AZ, GO, and GO-AZ on *C. albicans* yeast-to-hyphal morphology, a temporal observation of hyphae was examined using a microscope, whereas developed hyphae as a filamentous bunch were predominately seen after 24 h of incubation for nontreated yeast (Figure 3a). Interestingly, GO shows lower/delayed hyphal production at 2 µg/mL (Figure 3c). It is plausible that GO impacts the cell separation processes which caused polarized hyphal growth. However, further exploration is necessary to prove this hypothesis. On the other hand, presence of AZ (2 µg/mL) in GO-AZ more effectively suppressed the hyphae formation and cell aggregation (Figure 3d), which was similar to AZ (2 µg/mL) (Figure 3b), confirming that the dye-conjugated GO could prevent biofilm development as well as hyphal growth of *C. albicans*. Basically, biofilms were constructed by a highly structured crisscrossing hyphae to prevent the yeast from external toxic substances. From the phenotypic analysis, we realized that an anthraquinone derivative AZ with 2 hydroxyl groups could be a potent inhibitor of *C. albicans* filamentation, which matches with the previous report [34]. Perhaps not surprisingly, correlating hyphae filamentation and biofilm formation, subsequent experiments are in corroboration with the antibiofilm activity of AZ. Furthermore, AZ has been reported to reduce the hyphae-specific gene expressions and inhibition of endocytosis in the hyphae signaling pathway [34,41]. Although further investigations including, XTT (2,3-bis(2-methoxy-4-nitro-5-sulfophenyl)-2H-tetrazolium-5-carboxanilide sodium salt) reduction assays are necessary to elucidate the metabolic activities of yeast biofilm cells, GO-AZ composites would be worthy to consider as antibiofilm surface coating materials for further developments of fungus-free medical devices. 

### 3.4. Inhibition of *Candida* Virulence by GO-AZ in the Nematode Model

**Figure 4:**
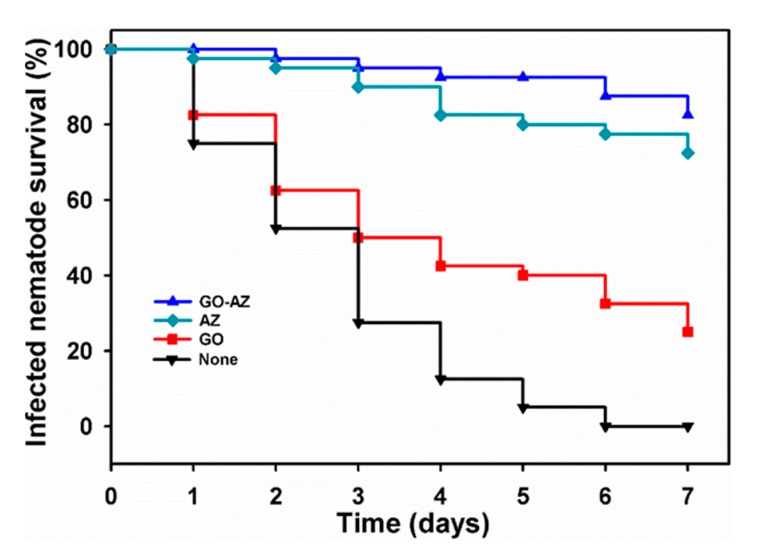
Kaplan–Meier survival curves of *C. albicans*-infected nematodes: Results representing the survivability of *C. elegans* with GO, AZ, and GO-AZ at 10 µg/mL. Nematodes without a sample treatment were considered as none. The percentage survivability exhibit the results of three independent experiments (n = 60) performed.

We explored the survivability of *C. albicans*-infected *C. elegans*, which have been considered as an alternative to mammalian model systems [42] by treating with AZ, GO, and GO-AZ at 10 µg/mL. As shown in Figure 4, the untreated nematode showed quick fatality and, at 6 days, complete killing was recorded because of *C. albicans* infection. At the end of 7 days, GO-treated nematode showed 20% survival against the infection (Figure 4), which was considered as less significant. Although GO at low concentration was nontoxic to the nematode [43,44], the fungi infection predominantly caused lethality to the majority of nematodes. However, almost 75% and more than 80% nematodes survived (Figure 4) in the presence of AZ and GO-AZ, respectively. These results clearly indicate that both AZ and GO-AZ showed no significant difference in nematode survivability, potentially diminished the Candida virulence in vivo, promoted the recovery of nematode, and prolonged the survival time of the infected nematode. Moreover, in comparison to GO toxicity, nematodes survived longer in the presence of AZ and GO-AZ, indicating that nontoxic AZ conjugation brings no toxicity. Hence, GO-AZ could be considered a potential platform for the development of medical devices which would provide a stable surface coating compared to free AZ with long-term antibiofilm action in the applied in vivo system. 

To the best of our knowledge, one-step conjugation of AZ on GO has never been experimented. Through this report, we claim a preparation of GO-AZ for the effective treatment of a highly virulent *C. albicans* biofilm even though the impact of AZ on *C. albicans* biofilm was explained previously [22] with a limited applicability of being employed as a biomedical devices surface coating material. Moreover, by this method, GO-AZ was produced in one step, with no additional chemicals, less processing time, and no toxicity and antibiofilm activity and could be utilized as a surface coating agent considering as a major advantage. Due to higher conjugation of AZ and immense surface-to-volume ratio of GO-AZ nanosheets, a minimal quantity of active chemical compound is sufficient to produce anticipated antibiofilm activity, whereas other methods require higher doses of payloads to achieve the same [10]. The significant antifungal properties of GO-AZ open a new window of hypothesis that this graphene oxide-containing alizarin composite could replace the existing coating material for biomedical devices with long-lasting antibiofilm activity. 

## 4. Conclusions

In summary, our findings suggest that prepared GO-AZ composite is a safe material, containing a potential therapeutic effect against disseminated candidiasis. The infected *C. elegans* was successfully treated within 7 days by GO-AZ with maintained survivability. This innovative application of preventing fungal cell adhesion and of creating a favorable surface for biofilm control makes the GO-AZ an attractive material for medical device coatings. Although the preliminary results are promising, further in vitro and in vivo investigations needed to be realized for the precise mechanism of GO-AZ on fungal cells. Future studies will be aimed to examine the composite efficiency on clinical species involved in polymicrobial medical devices infection.
